# Advances in clinical research on interstitial lung disease

**DOI:** 10.3389/fmed.2025.1714301

**Published:** 2025-12-04

**Authors:** Xiao-Lian Zhou, Xiao-Bo Hu, Yong-Sheng Wang, Duo Li

**Affiliations:** 1 Department of Pulmonary and Critical Care Medicine, West China School of Medicine, Sichuan University, Sichuan University affiliated Chengdu Second People’s Hospital, Chengdu Second People’s Hospital, Chengdu, Sichuan, China; 2 Department of Pulmonary and Critical Care Medicine, The Affiliated Hospital of Southwest Medical University, Luzhou, Sichuan, China; 3 Department of Infection Management, The Affiliated Hospital of Southwest Medical University, Luzhou, Sichuan, China

**Keywords:** interstitial lung disease, pathogenesis, biomarkers, precision medicine, cutting-edge progress

## Abstract

Interstitial lung disease (ILD) is a group of pulmonary disorders characterized by complex etiologies and diverse clinical manifestations, with an increasing incidence trend. This article aims to review the cutting-edge research on ILD, including new insights into its pathogenesis, advances in diagnostic technologies, and innovations in therapeutic strategies. It deeply explores the key roles of signaling pathways such as TGF-β and Wnt/β-catenin in pulmonary fibrosis, as well as the mechanisms of alveolar epithelial cell injury and repair. Meanwhile, it details the applications of novel biomarkers (e.g., blood, respiratory tract, and imaging biomarkers) in disease diagnosis and condition monitoring. In terms of treatment, it elaborates on the research progress in frontier directions such as clinical trials of new drugs, precision medicine and individualized treatment, and cell therapy and regenerative medicine, in order to provide the latest theoretical basis and diagnostic/therapeutic ideas for clinical practice.

## 1 Introduction

Interstitial lung disease (ILD) is not a single disease, but a collection encompassing more than 200 distinct types of lung diseases. Their common characteristic is that the pathological changes mainly involve the lung interstitium, leading to damage of the alveolar-capillary functional unit. This, in turn, causes progressive dyspnea and restrictive ventilatory dysfunction, and usually results in end-stage respiratory failure (1). Currently, epidemiological data on pulmonary interstitial fibrosis mainly come from developed countries such as those in Europe and North America, as well as some coastal cities in Asia. The average age of patients with pulmonary fibrosis is approximately 65–70 years old, and the incidence increases with age. Pulmonary fibrosis affects more men than women. A large number of studies have shown that risk factors such as smoking, inhalation of metals/wood dust, and genetic factors have been confirmed to be associated with its development (2). Interstitial pneumonia is a major type of interstitial lung disease. Selman et al. argued that unifying usual interstitial pneumonia (UIP) as a single, independent diagnostic entity has significant advantages, integrating its primary forms and secondary processes into diseases such as hypersensitivity pneumonitis and rheumatoid arthritis-associated interstitial lung disease (3). In the ERS/ATS (European Respiratory Society/American Thoracic Society) statement, Ryerson et al. based on the 2013 classification of interstitial pneumonia, new interstitial pneumonia subcategories were added: bronchiocentric interstitial pneumonia, alveolar macrophage pneumonia, and idiopathic diffuse alveolar damage (4). Among all interstitial lung diseases, progressive fibrosing interstitial lung diseases (PF-ILDs) progress rapidly and have an extremely high mortality rate. Rajan et al. in their expert consensus statement, emphasized the importance of early identification of patients with progressive pulmonary fibrosis, accurate risk stratification, and the development of personalized monitoring strategies for managing the risk of progression (e.g., guidance on pulmonary rehabilitation, oxygen therapy, and the use of non-invasive ventilation) (5, 6). The etiology of ILD is complex. With the continuous advancement of medical research, a series of important progress has been made in recent years in the pathogenesis, diagnosis, and treatment of ILD.

## 2 New insights into pathogenesis

### 2.1 The role of signaling pathways in pulmonary fibrosis

#### 2.1.1 TGF-β signaling pathway

Similar to the pathophysiology of lung cancer brain metastasis, cancer cells participate in cascade reactions, and multiple factors such as cytokines, adhesion molecules, and gene activity regulate the migration, growth, and invasion of tumor cells (7). In pulmonary fibrosis, transforming growth factor-β1 (TGF-β1) is widely recognized as playing a central role in the progression of pulmonary fibrosis. TGF-β1 is the most potent mediator in the pathogenesis of pulmonary fibrosis (PF), which mediates the Smad pathway and exerts a crucial effect on the development of fibrosis. TGF-β1 binds to type I and type II TGF-β receptors to form an activated kinase domain, leading to the phosphorylation of Smad2/3. Phosphorylated Smad2/3 forms a heterotrimeric complex with Smad4, which then undergoes nuclear translocation. This process induces the expression of microRNAs (miRNAs) and inhibits epithelial cell marker proteins, thereby effectively promoting alveolar epithelial-mesenchymal transition (EMT). Once translocated into the nucleus, the heterotrimeric complex can regulate the expression of various genes–including collagen, α-smooth muscle actin (α-SMA), and connective tissue growth factor (CTGF)–by directly binding to their promoters, thus facilitating the occurrence of pulmonary fibrosis (8). In addition, TGF-β1 can also synergistically promote the development of pulmonary fibrosis by regulating other cytokines and signaling pathways. A study by Gao et al. found that pyruvate kinase M2 (PKM2) promotes fibrotic progression by directly interacting with Smad7 and enhancing TGF-β1 signaling (9).

#### 2.1.2 Wnt/β-catenin signaling pathway

The Wnt/β-catenin signaling pathway plays a crucial regulatory role in cell proliferation, differentiation, and tissue morphogenesis. In recent years, studies have found that this signaling pathway also plays an important role in the pathogenesis of ILD. During the process of pulmonary fibrosis, the Wnt signaling pathway is abnormally activated, leading to the accumulation of β-catenin in the cytoplasm and its subsequent entry into the nucleus. Once in the nucleus, β-catenin binds to transcription factors, regulates the expression of a series of target genes, and promotes the activation and proliferation of fibroblasts. Meanwhile, the Wnt/β-catenin signaling pathway can also interact with the TGF-β signaling pathway, jointly promoting the progression of pulmonary fibrosis (10).

### 2.2 Mechanism of alveolar epithelial cell injury and repair

Alveolar epithelial cells play a key role in maintaining the normal structure and function of the lungs. During the occurrence and development of ILD, alveolar epithelial cells are often damaged by various pathogenic factors, leading to apoptosis or necrosis. This damage impairs the alveolar barrier function, triggers the infiltration of inflammatory cells and the release of cytokines, and initiates the process of pulmonary fibrosis (11). In the early stage of injury, type II alveolar epithelial cells can proliferate and differentiate into type I alveolar epithelial cells to repair the damaged alveolar epithelium (12).

### 2.3 Immunoregulatory mechanisms

**FIGURE 1:**
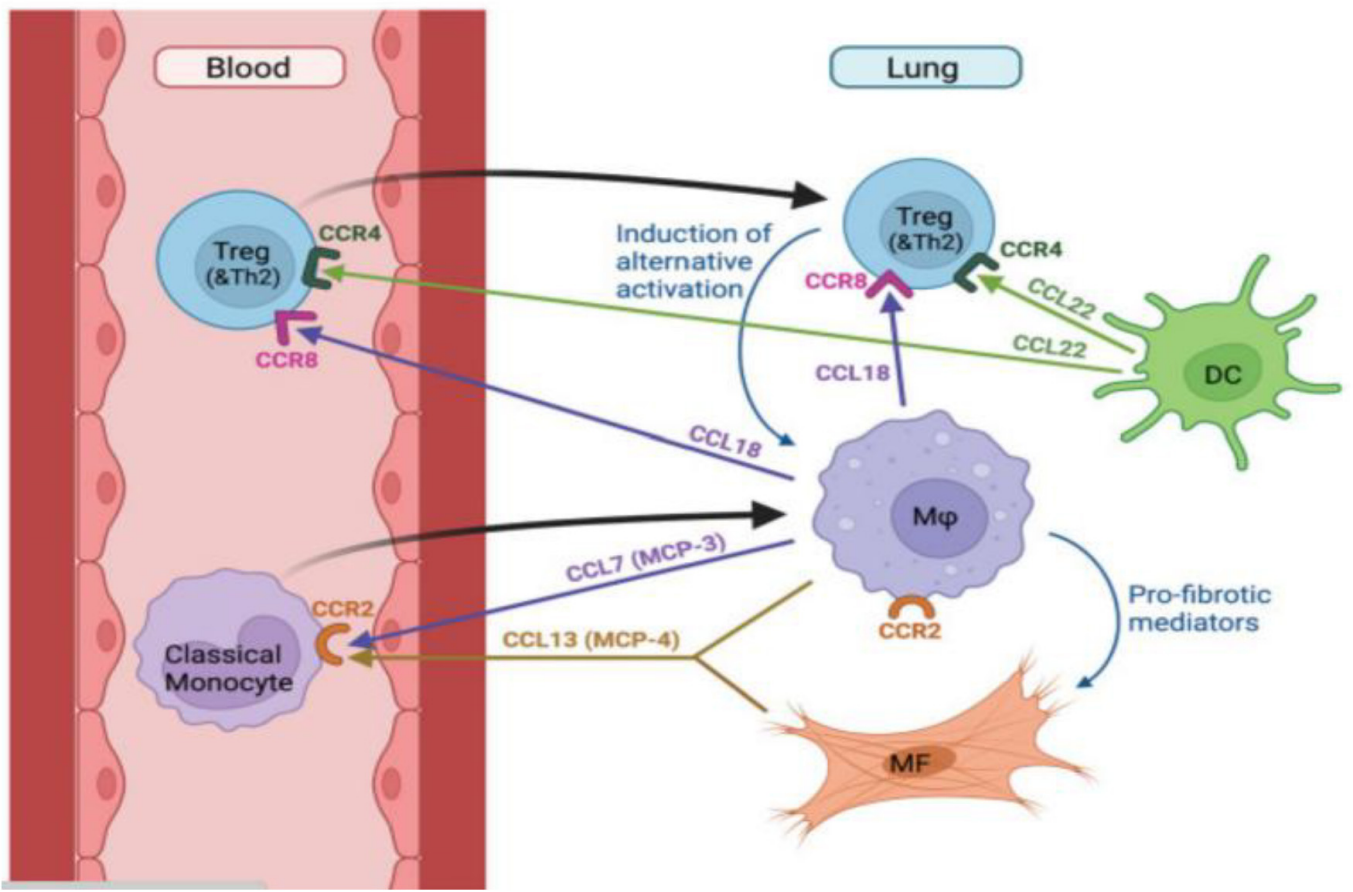
The lung-blood recruitment model in pulmonary fibrosis. Figure 1 was created using BioRender software.

The immune system plays a crucial regulatory role in the pathogenesis of ILD. The number of macrophages increases in the lung tissue of patients with ILD, and these cells can secrete a variety of cytokines and inflammatory mediators, such as tumor necrosis factor-α (TNF-α), interleukin-1 (IL-1), and interleukin-6 (IL-6). These substances trigger and amplify inflammatory responses in the lungs (13). Unterman et al. proposed a lung-blood recruitment model in progressive pulmonary fibrosis (see Figure 1). Pulmonary macrophages and dendritic cells (DCs) secrete CCL18 and CCL22, respectively. These chemokines may drive the recruitment of regulatory T cells (Treg) and T helper 2 cells (Th2) mediated by CCR8 and CCR4, thereby promoting pulmonary fibrosis (14). Mehta et al. confirmed the presence of profibrotic macrophages through bronchoscopy in patients with COVID-19, and these profibrotic macrophages are associated with the pathogenesis of severe COVID-19-associated acute respiratory distress syndrome (ARDS) (15). Additionally, Katsura et al. proposed that lung stem cells can indirectly delay the progression of fibrosis through their immunoregulatory capabilities (16).

## 3 Advances in diagnostic technologies

### 3.1 Application of diagnostic tools

In recent years, diagnostic tools for ILD have been continuously updated. Common diagnostic tools include high-resolution computed tomography (HRCT), transbronchial lung cryobiopsy (TBLC), surgical lung biopsy (SLB), and endobronchial ultrasound-guided cryobiopsy (EBUS-C). Multiple studies have shown that lung ultrasound, exhaled breath analysis, and genomic classifiers (GC) also hold significant diagnostic value in interstitial lung diseases. For details, see Table 1 on Diagnostic Tools (17).

**TABLE 1:** Content, advantages and disadvantages of ILD diagnostic tools.

Diagnostic tool	Specific content	Advantages	Limitations
High-resolution CT (HRCT)	Non-invasively visualizes fine lung structures and guides diagnostic direction	Non-invasive; clarifies lesion characteristics and distribution	Cannot confirm pathological diagnosis; involves radiation
Lung ultrasound (LUS)	Bedside assessment and dynamic monitoring	Non-invasive; convenient	Low diagnostic specificity; susceptible to interference
Bronchoscopy	Bronchoalveolar lavage (BAL), Transbronchial lung cryobiopsy (TBLC), Endobronchial ultrasound-guided cryobiopsy (EBUS-C)	High diagnostic rate	High risk
Surgical lung biopsy (SLB)	Video-assisted thoracoscopic surgery (VATS) lung biopsy and open thoracotomy lung biopsy (OTLB)	Extremely high accuracy; potential therapeutic value	Severe trauma; high risk
Genomic classifier (GC)	Excellent specificity for UIP (approximately 90%)	Non-invasive; low risk	Cannot diagnose specific patterns
Exhaled breath analysis	Electronic nose (eNose) sensor technology	Non-invasive; rapid	Not yet widely used in clinical practice
Quantitative imaging analysis (QIA)	Enables quantification for the diagnosis of interstitial lung abnormality (ILA) or ILD	Objective; quantitative	Currently relatively limited in application scope

#### 3.1.1 High-resolution computed tomography (HRCT)

High-resolution computed tomography is currently the most important imaging modality for diagnosing ILD. In recent years, HRCT technology has continued to advance; for example, the adoption of low-dose scanning technology not only ensures image quality but also reduces the patient’s radiation exposure. HRCT can detect characteristic fibrotic changes, such as reticulation (reticular patterns), interlobular septal thickening, or “honeycombing” patterns (18). Additionally, computer-aided diagnosis (CAD) technology enhances the accuracy of identifying ILD-related imaging features, which is conducive to early diagnosis and disease monitoring (19).

#### 3.1.2 Positron emission tomography (PET)

Positron emission tomography scanning helps assess the activity of ILD and treatment response by detecting tissue metabolic activity. In patients with ILD, the metabolic activity in areas of pulmonary lesions is often increased, and PET scans can visualize corresponding hypermetabolic foci (20). Combining PET with technologies such as computed tomography (CT) or magnetic resonance imaging (MRI) enables simultaneous acquisition of metabolic information and anatomical structural information of lesions, further improving the ability to diagnose and evaluate ILD.

#### 3.1.3 Lung ultrasound

A prospective study conducted by Vassalou et al. showed that lung ultrasound in the lateral decubitus position is more convenient and rapid for diagnosing idiopathic pulmonary fibrosis (IPF). Compared with the sitting and supine positions in patients, it has a higher correlation with high-resolution computed tomography (HRCT) (21). Moreover, a study by Pitsidianakis et al. found that lung ultrasound can also monitor the progression of interstitial lung disease (ILD) (22). A recent systematic literature review has confirmed that lung ultrasound holds significant diagnostic value in evaluating ILD in systemic sclerosis and other musculoskeletal diseases characterized by ILD (23).

#### 3.1.4 Exhaled breath analysis

A study by Moor et al. revealed that electronic nose (eNose) technology can completely distinguish ILD patients from healthy controls and accurately differentiate between different ILD subgroups. Exhaled breath analysis using eNose technology serves as an early diagnostic tool for ILD (24).

### 3.2 Application of biomarkers

#### 3.2.1 Blood biomarkers

The detection of blood biomarkers provides a convenient method for the diagnosis and disease monitoring of ILD. Currently, blood biomarkers that have been extensively studied include KL-6, surfactant protein D (SP-D), monocyte count, matrix metalloproteinases (MMPs), and their tissue inhibitors (TIMPs). KL-6 is a mucin-like glycoprotein; its level in the serum of ILD patients is significantly elevated, and the degree of elevation is positively correlated with disease severity. Thus, KL-6 can serve as a crucial indicator for ILD diagnosis, disease monitoring, and prognostic evaluation (25). Through a retrospective study, Kreuter et al. found that monocyte count may provide a simple prognostic biomarker for idiopathic pulmonary fibrosis (IPF). In IPF patients, an elevated monocyte count may indicate IPF progression. Compared with the Fibrosis Severity Index, monocyte count can identify patients with fibrotic diseases at high risk of death earlier and is associated with an increased risk of death (26, 27).

#### 3.2.2 Respiratory biomarkers

The analysis of inflammatory biomarkers in exhaled breath condensate (EBC) offers a non-invasive auxiliary method for ILD diagnosis (28). EBC contains a variety of inflammatory mediators, such as 8-isoprostane F2α (8-iso-PGF2α) and nitric oxide (NO). The fractional concentration of nitric oxide in exhaled air from the lower respiratory tract (CaNO) in ILD patients is higher than that in healthy individuals, indicating that inducible nitric oxide synthase (iNOS) is activated during the stage of pulmonary fibrosis involvement. This activation promotes NO production, further exacerbating pulmonary inflammation and fibrosis (29).

### 3.3 Pulmonary function testing

Pulmonary function testing is a key method for evaluating the degree and type of pulmonary function impairment in ILD patients. ILD patients mainly present with restrictive ventilatory dysfunction, characterized by decreased vital capacity (VC) and total lung capacity (TLC), while the ratio of forced expiratory volume in 1 s (FEV1) to forced vital capacity (FVC) remains normal or increases. Lu et al. found through research that nearly 40% of IPF patients exhibit small airway disease in the early stage. Forced oscillation technique (FOT) should be introduced to detect small airway abnormalities in IPF at an early stage, as these abnormalities are associated with poor prognosis and increased risk of death (30). In a study, Chang et al. discovered that elevated disease activity of rheumatoid factor in patients with rheumatoid arthritis (RA) is closely related to airway abnormalities. Investigating the prevalence of airway abnormalities through pulmonary function testing is of great significance for the early diagnosis of IPF (31).

### 3.4 Genetic testing

Genetic testing plays an increasingly important role in the diagnosis and research of ILD. For ILD patients with familial aggregation, genetic testing helps identify the etiological cause and provides a basis for genetic counseling and precision treatment (32). For instance, gene mutations in telomerase reverse transcriptase (TERT) and telomerase RNA component (TERC) are associated with familial pulmonary fibrosis; detecting these gene mutations can assist in the diagnosis of hereditary pulmonary fibrosis.

## 4 Innovations in therapeutic strategies

### 4.1 Clinical trials of novel drugs

#### 4.1.1 Antifibrotic drugs

Currently, antifibrotic drugs are one of the important therapeutic approaches for ILD. In addition to the marketed pirfenidone and nintedanib, a variety of novel antifibrotic drugs are undergoing clinical trials. For example, *in vitro* experiments have confirmed that miR-182-5p is induced by TGF-β1 and has the function of promoting fibrosis. In dual-luciferase reporter gene assays, Smad7 was shown to be negatively regulated by miR-182-5p; therefore, inhibiting miR-182-5p can be regarded as an effective method for treating idiopathic pulmonary fibrosis (IPF) (33). Furthermore, antifibrotic drugs targeting other signaling pathways or molecular targets–such as Wnt signaling pathway inhibitors and connective tissue growth factor (CTGF) inhibitors–are also in the research stage (34).

#### 4.1.2 Immunomodulators

Given the important role of the immune system in the pathogenesis of ILD, immunomodulators have also become a research focus in the treatment of ILD. In recent years, novel immunomodulators have emerged continuously, such as Janus kinase (JAK) inhibitors. JAK inhibitors regulate immune responses and alleviate pulmonary inflammation by inhibiting cytokine signal transduction. JAK2 is highly expressed in proliferative alveolar epithelial cells and fibroblast-like cells, and the JAK2 signaling pathway also plays a key role in idiopathic pulmonary fibrosis (IPF) (35). In clinical trials, JAK inhibitors have shown good efficacy in some ILD patients; two inhibitors, pracinostat and panobinostat, can exert antifibrotic effects by inhibiting the JAK2 signaling pathway, with significant results (36).

### 4.2 Precision medicine and individualized treatment

Interstitial lung disease exhibits high heterogeneity, with significant differences in etiology, pathological type, clinical manifestations, and treatment response among different patients. Therefore, the concepts of precision medicine and individualized treatment have received increasing attention in ILD treatment. Developing individualized treatment plans can improve therapeutic efficacy and safety (37). Bermudo et al. and Spagnolo et al. have mentioned in their studies the necessity of primary care for the early and accurate diagnosis of ILD patients. There are four types of patients suspected of having ILD in primary care settings: subclinical cases refer to patients without respiratory symptoms, and patients at risk of developing ILD. Chest X-rays, computed tomography (CT) scans, and spirometry are helpful for assisting in the diagnosis of ILD in primary care centers (38, 39).

The Clinical Frailty Scale (CFS) can be used for risk stratification of ILD patients; it is associated with the decline in lung function and physical function in ILD patients, which improves the prognostic accuracy in clinical practice (40). Kirsten et al. proposed a questionnaire for assessing the quality of life in idiopathic pulmonary fibrosis (Quality of Life in Pulmonary Fibrosis Questionnaire, QPF), which is suitable for screening the quality of life of idiopathic pulmonary fibrosis patients and monitoring the course of their disease (41). Multiple studies have shown that patient-centered care tailored to different individuals is extremely important for the management of patients with respiratory failure related to pulmonary fibrosis (42). Patients with pulmonary fibrosis often experience depression and anxiety; in addition to effective medications, attention should also be paid to psychosocial interventions and mental health support for patients (43).

### 4.3 Cellular therapy and regenerative medicine

#### 4.3.1 Stem cell therapy

Stem cells possess self-renewal and multi-directional differentiation potential, and exhibit great promise in tissue repair and regeneration. Mesenchymal stem cells (MSCs) have become a research focus in ILD treatment due to their properties such as immunomodulation, anti-inflammation, and promotion of tissue repair. Zhou et al. reported a successful case of treating anti-MDA5 antibody-positive dermatomyositis complicated with interstitial lung disease using human umbilical cord mesenchymal stem cells (44). By secreting a variety of cytokines and growth factors, MSCs can regulate immune responses, inhibit inflammatory cell infiltration, promote the repair of alveolar epithelial cells and vascular endothelial cells, and reduce pulmonary fibrosis.

#### 4.3.2 Alveolar epithelial cell regeneration

Promoting alveolar epithelial cell regeneration is another important research direction for ILD treatment. As mentioned earlier, abnormal injury and repair of alveolar epithelial cells play a key role in the pathogenesis of ILD. By investigating the molecular mechanisms of alveolar epithelial cell regeneration and developing therapeutic approaches that promote the proliferation and differentiation of alveolar epithelial cells, it is expected to repair damaged alveolar structures and improve lung function (45). In addition, tissue engineering techniques can also be used to construct artificial alveolar epithelial tissue for repairing damaged lung tissue–for example, in 3D bioprinting, biomaterials function as the extracellular matrix (ECM) for cells, providing sufficient mechanical support and facilitating cell connection to shape cells and repair tissues (46). Although alveolar epithelial cell regeneration therapy is still in the research stage, it brings new hope for the treatment of ILD.

### 4.4 Comprehensive treatment strategies

The treatment of ILD often requires the integration of multiple therapeutic approaches to improve treatment efficacy. In addition to drug therapy and cellular therapy, attention should also be paid to supportive care and rehabilitation training for patients. For ILD patients, nutritional support should be provided to ensure adequate intake of nutrients such as protein. Furthermore, psychological support and psychological intervention can help patients regain confidence and cooperate with treatment. Comprehensive treatment strategies can improve the condition of ILD patients in multiple aspects and enhance their quality of life (47).

## 5 Summary and outlook

As a group of complex and heterogeneous lung diseases, interstitial lung disease (ILD) involves multiple aspects in its pathogenesis, including abnormal signaling pathways, alveolar epithelial cell injury, and immunoregulatory disorders. In recent years, innovations in imaging technology, the application of biomarkers, and the popularization of genetic testing have significantly improved the diagnostic accuracy and early identification ability of ILD; the exploration of novel antifibrotic drugs, immunomodulators, and cellular therapy has provided more treatment options for patients. However, the field of ILD still faces challenges such as difficult etiological diagnosis, limited treatment efficacy, and significant heterogeneity. In the future, through the innovation of precise diagnostic technologies, the development of targeted therapeutic drugs, breakthroughs in cellular therapy and regenerative medicine, and the innovation of disease management models, it is expected to gradually solve the existing problems and further promote the improvement of ILD diagnosis and treatment.
